# Sex and age difference in risk factor distribution, trend, and long-term outcome of patients undergoing isolated coronary artery bypass graft surgery

**DOI:** 10.1186/s12872-021-02273-2

**Published:** 2021-09-23

**Authors:** Babak Sattartabar, Ali Ajam, Mina Pashang, Arash Jalali, Saeed Sadeghian, Hamideh Mortazavi, Soheil Mansourian, Jamshid Bagheri, Abbas-Ali Karimi, Kaveh Hosseini

**Affiliations:** 1 Department of Cardiology, Tehran Heart Center, Tehran University of Medical Sciences, North Kargar Ave., 1411713138 Tehran, Iran; 2 Research Department, Tehran Heart Center, Tehran University of Medical Sciences, Tehran, Iran; 3 Students’ Scientific Research Center (SSRC), Tehran University of Medical Sciences, Tehran, Iran; 4 Department of Cardiac Surgery, Tehran Heart Center, Tehran University of Medical Sciences, Tehran, Iran

**Keywords:** Coronary artery bypass graft surgery, Cardiovascular risk factor, Inverse probability weighting

## Abstract

**Background:**

Preoperative coronary artery disease risk factors (CADRFs) distribution and pattern may also have an important role in determining major adverse cardiovascular events (MACEs). In this study, we aimed to evaluate the CADRFs distribution and trend over 10 years and also the long-term outcome of CABG in different age-sex categories.

**Method:**

In this registry-based serial cross-sectional study, we enrolled 24,328 patients who underwent isolated CABG and evaluated the prevalence of CADRFs according to sex and age. We used inverse probability weighting (IPW) to compare survival and MACE between the sexes. We also used Cox regression to determine each CADRFs effect on survival and MACEs.

**Results:**

In general, DLP (56.00%), HTN (53.10%), DM (38.40%), and positive family history (38.30%) were the most frequent risk factors in all patients. Prevalence of HTN, DLP, DM, obesity, and positive family history were all higher in women, all statistically significant. The median follow-up duration was 78.1 months (76.31–79.87 months). After inverse probability weighting (to balance risk factors and comorbidities), men had lower MACEs during follow-up (HR 0.72; 95% CI 0.57–0.91; *P* value 0.006) and there was no significant difference in survival between sexes. DM and HTN were associated with higher mortality and MACEs in both sexes.

**Conclusion:**

Although DLP is still the most frequent CADRF among the CABG population, the level of LDL and TG is decreasing. Women experience higher MACE post CABG. Therefore, health care providers and legislators must pay greater attention to female population CADRFs and ways to prevent them at different levels.

## Introduction

Coronary artery disease (CAD) is a chronic, progressive, multifactorial disease that is the leading cause of morbidity and mortality worldwide, especially in developing countries [1, 2]. Coronary artery bypass grafting (CABG) has been an exquisite treatment for complex CAD since the 1960s [3]. Despite the increasing rate of percutaneous coronary intervention (PCI), CABG is still the most prevalent cardiac surgery [4].

CAD’s risk factors such as hypertension (HTN), diabetes mellitus (DM), dyslipidemia (DLP), cigarette smoking, and family history of CAD were introduced by the Framingham study [5]. Sex and Age are among the most important non-modifiable risk factors, for example, CAD develops in women later in life comparing to men for an unknown reason [6]. Late-onset of CAD in women results in underestimation of cardiovascular risk by healthcare providers and patients [7] and leads to a higher burden of CAD in women, especially at a young age.

Although surgical techniques in CABG improved in recent years, there is still a chance for adverse outcomes, in which pre-operative modifiable and non-modifiable risk factors play an important role. Preoperative coronary artery disease risk factors (CADRFs) distribution and pattern may have an essential role in determining major adverse cardiovascular events (MACEs) and survival after surgery. The impact of CADRFs such as age (premature vs. older age) and sex in CAD is not distinguished [7–9].

Hence, in this article, we aimed to evaluate the following subjects: A. CADRFs distribution and trend over 10 years among isolated CABG patients; B. Sex difference in CADRFs distribution and C. Post-CABG survival and MACE in the whole population and also in premature population.

## Methods

### Tehran heart center

Tehran heart center (THC) is a major tertiary referral center in Tehran, Iran, dedicated to the treatment of heart diseases. From 2001 to the end of 2017, approximately 1,300,000 patients were referred to the outpatient clinics, more than 800,000 patients visited the emergency rooms, over 280,000 patients were hospitalized, and more than 55,000 patients underwent open-heart surgeries THC. Additionally, more than 150,000 coronary angiographies and over 35,000 angioplasties were done in this center.

Data regarding risk factors were collected in THC clinics, including the risk factors clinic, open heart surgery clinic, and angioplasty clinic. The risk factors clinic was established in 2005 to control the CAD risk factors for all patients. Patients who undergo either coronary artery angioplasty or open-heart surgery receive additional follow-up in the angioplasty follow-up clinic for 3–5 years or the open-heart surgery clinic for up to 10 years [10].

### Coronary angiography data bank

The coronary angiography data bank, established in January 2005, registers collected data from all patients who undergo angiography at THC. As part of the THC protocol, demographic, clinical, and laboratory data are collected during the admission to the catheterization laboratory and are recorded in an electronic registry. Informed consent is required by patients for inclusion in the data bank.

### Design and participants

In this single-center registry-based serial cross-sectional study, all patients in the coronary angiography data bank of the THC from 2007 through 2017 were reviewed. Patients with obstructive CAD (defined by an angiography report of more than 50% stenosis in at least one vessel) were eligible. We excluded patients with implausible data which is defined as following, age younger than 20 or older than 100, fasting blood sugar less than 50 or higher than 600, LDL cholesterol of less than 20 or higher than 500, HDL cholesterol of less than 5 or greater than 100, total cholesterol of less than 50 or greater than 700, triglyceride level of less than 20 or greater than 1200, and creatinine level of less than 0.2 or greater than 10. The prevalence of conventional CAD risk factors including hypertension (HTN), dyslipidemia (DLP), diabetes mellitus (DM), obesity, cigarette smoking, and family history of CAD were analyzed according to sex and age, and the 10-year trends were demonstrated.

### Definitions and protocols

HTN was defined by at least two office blood pressure (BP) measurements exceeding 140 over 90 mmHg. Blood pressure was measured using a manual mercury sphygmomanometer in all cases. Trained nurses measured blood pressure in both hands in a sitting position after resting for 5 min. The higher BP was registered in a pre-specified datasheet. This protocol was repeated after 3 min. In case of difference (more than 10 mmHg in systolic blood pressure of 5 mmHg in diastolic blood pressure), the measurement was repeated for the third time and the two measurements closer together were used. The mean systolic and diastolic blood pressure of the two measurements was calculated. Participants with high blood pressure were defined as follows: mean systolic blood pressure ≥ 140 mmHg or mean diastolic blood pressure of ≥ 90 mmHg or who were taking blood pressure medications. DM was defined by measurements of fasting plasma glucose ≥ 126 mg/dL or glycated hemoglobin A1C (HbA1C) ≥ 6.5% in the presence of confirmatory testing. Patients on antidiabetic medications were also included as diabetic in the study. Determination of DLP was according to either total serum cholesterol ≥ 200 mg/dL, high-density lipoprotein cholesterol < 40 mg/dL in men and < 50 mg/dL in women, or triglycerides ≥ 250 mg/dL, measured after at least ten hours of fasting. Patients with a previous diagnosis of DLP who were on lipid-lowering agents were also included. Obesity was considered in patients who had a body mass index (BMI) ≥ of 30 kg/m^2^. The definition of smoking was having ever smoked more than 100 cigarettes. Family history was considered in the presence of atherosclerotic cardiovascular disease (ASCVD) in first-degree male relatives aged < 55 years or in females aged < 65 years. Premature CAD was defined as the occurrence of CAD in men under 50 and women under 55 [11]. We defined MACE as cardiovascular death, myocardial infarction, or ischemic stroke.

### Statistical analysis

Categorical variables are presented as numbers (percentage) and compared using the Chi-square test. Numerical variables were demonstrated as mean ± SD and compared using a T-test. Categorical variables were described as absolute frequencies and compared between age groups by the χ^2^ and Fisher exact tests, as appropriate. The median follow-up time was calculated via the inverse Kaplan–Meier method. Cox proportional hazards models were used to estimate the unadjusted and adjusted effects of variables on all-cause mortality and MACCE. abovementioned outcomes were adjusted for age, sex, diabetes mellitus (DM), hypertension (HTN), dyslipidemia (DLP), the body mass index (BMI), family history of premature CAD, cigarette smoking (CS), opium consumption, the ejection fraction, a previous percutaneous coronary intervention, left main coronary artery stenosis exceeding 50%, history of MI, renal failure, chronic obstructive pulmonary disease, cerebrovascular accidents, the type of surgery (off-pump or on-pump), the status of surgery (urgent and emergent), and the number of risk factors. Comparison between male and female populations was done via the stabilized IPW method. Weights were calculated from propensity scores derived from the predicted probabilities of the logistic regression of women versus men on identified potential confounders. All the statistical analyses were conducted applying IBM SPSS Statistics for Windows, version 23.0 (Armonk, NY: IBM Corp), and Stata Statistical Software: Release 14 (College Station, TX: StataCorp LP).

## Result

### Population

During this study, we evaluated 24,328 patients who underwent CABG in THC from 2007 to 2017. The mean age was 60.8 ± 9.52, 6428 (26.4%) were women and 17,900 (73.6%) were men. The mean age of patients increased from 60.7 ± 9.46 to 61.7 ± 9.5 and the male to female ratio remained nearly constant at approximately 2.8 (Table 1). The median follow-up duration was 78.1 months (76.31 to 79.87 months).

**Table 1:** Patients’ demographic characteristics in each 3 years

Year	Age	Sex (%)	Coronary artery disease risk factors						
			HTN	DLP	DM	Cigarette smoking	Obesity	Opium addiction	Family History
2007–2010	60.7 ± 9.46	Male 73.2	5210 (51.90%)	6201 (61.70%)	3491 (34.70%)	1645 (16.40%)	2361 (23.6%)	1122 (11.40%)	4278 (42.60%)
		Female 26.8							
2011–2013	60.81 ± 9.53	Male 73.3	2945 (48.30%)	3003 (49.20%)	2483 (40.70%)	961 (15.80%)	1366 (22.4%)	629 (10.80%)	2594 (42.50%)
		Female 26.7							
2014–2017	61.7 ± 9.5	Male 74.3	4760 (58.40%)	4387 (53.90%)	3363 (41.20%)	1656 (20.40%)	2017 (24.9%)	1509 (18.60%)	2449 (30.00%)
		Female 25.7							
Total	60.8 ± 9.52	Male 73.6	12,915 (53.10%)	13,591 (56.00%)	9337 (38.40%)	4262 (17.60%)	5744 (23.7%)	3260 (13.70%)	9321 (38.30%)
		Female 26.4							

Data are presented as mean ± SD and number (%)

HTN: hypertension, DLP: dyslipidemia, and DM: diabetes mellitus

### Risk factor distribution

#### Total population

**Fig. 1:**
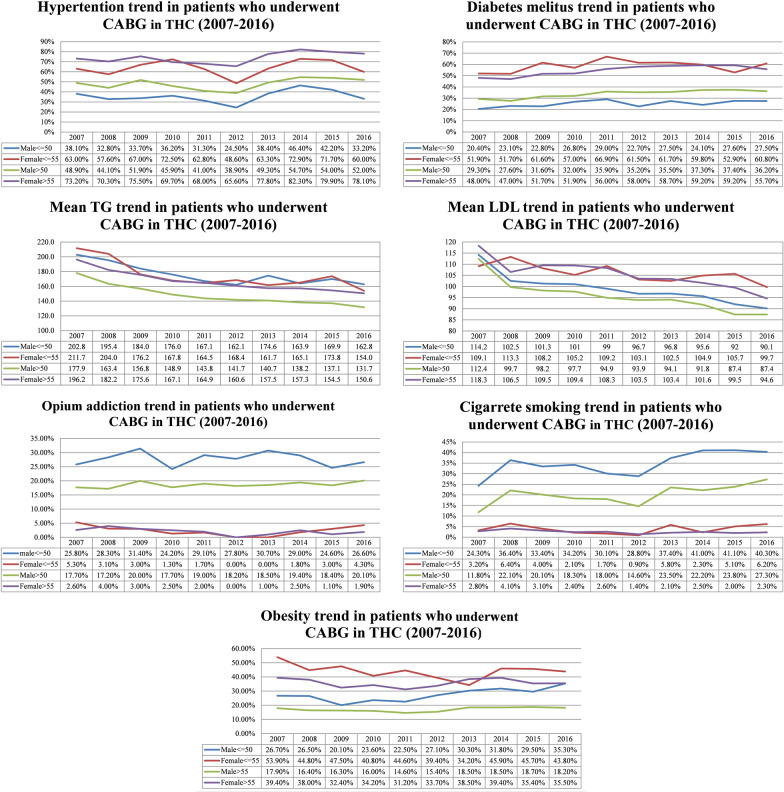
Trends of coronary artery disease risk factors after coronary artery bypass grafting among different age-sex groups; 2007–2016

We compared risk factors distribution in 3 time periods, 2007–2010, 2011–2013, and 2014–2017. In all of these periods, HTN and DLP were the most prevalent risk factors, present in more than half of the patients. Although not at a steady pace, HTN, DM, cigarette smoking, opium addiction tended to increase over the years. On the other hand, positive family history decreased significantly in the last period. As for DLP, despite a significant decrease in 2011–2013, it showed a slight increase in 2014–2017. Obesity remains nearly constant through the years being present in nearly a quarter of patients. In general, DLP (56.00%), HTN (53.10%), DM (38.40%), and positive family history (38.30%) were the most frequent risk factors in all patients (Table 1, Fig. 1).

#### Premature population, men versus women

Based on the age of premature CAD in men and women, risk factors distribution was categorized into 4 groups, men ≤ 50 (2883; 11.9%), men > 50 (15,018; 61.7%), women ≤ 55 (1448; 6.0%) and women > 55 (4979; 20.4%).

In premature CAD in men, DLP and positive family history were the most frequent risk factors, with DM and opium addiction being the least frequent ones. In men older than 50, DLP then HTN had the highest, and opium addiction and cigarette smoking had the lowest prevalence. Positive family history showed the biggest difference between the men of two age groups (52.6% in men ≤ 50 vs. 33.2% in men > 50). In men of all ages, DLP (51.3%) and HTN (46.5%) were the most frequent risk factors. In women ≤ 55, DLP, HTN, DM, and positive family history were all present in more than 50% of patients being the most prevalent risk factors respectively. HTN, DLP, and DM had the highest prevalence in women > 55. Similar to men, positive family history prevalence had the highest difference between women of the age groups. HTN (72.3%), DLP (69.7%), and DM (55.4%) were the most prevalent risk factors in all women. In all age-sex groups, DLP was the most frequent risk factor except for women > 55 whose HTN was slightly higher than DLP. Both TG and LDL had a nearly declining trend in 2007–2016, with women ≤ 55 having the highest levels and men over 50 having the lowest levels. In comparison between men and women, HTN, DLP, DM, Obesity, and positive family history were all higher in women, all statistically significant. Cigarette smoking and opium addiction were significantly lower in the female group, present in less than 5% of the patients despite increasing prevalence over the years (Table 2, Fig. 1).

**Table 2:** Risk factors distribution in each age-sex group

Age-sex category	Coronary artery disease risk factors						
	Hypertension	Dyslipidemia	Diabetes mellitus	Cigarette smoking	Obesity	Opium addiction	Family History
Men ≤ 50	1023 (35.50%)	1620 (56.30%)	708 (24.60%)	998 (34.60%)	775 (27.0%)	734 (25.90%)	1517 (52.60%)
Men > 50	7269 (48.50%)	7513 (50.10%)	5081 (33.90%)	3080 (20.60%)	2562 (17.1%)	2394 (16.40%)	4986 (33.20%)
Women ≤ 55	930 (64.30%)	1008 (69.70%)	844 (58.30%)	56 (3.90%)	646 (44.7%)	44 (3.00%)	803 (55.50%)
Women > 55	3693 (74.30%)	3450 (69.50%)	2704 (54.40%)	128 (2.60%)	1761 (35.6%)	88 (1.80%)	2015 (40.50%)
Men	8292 (46.4%)	9133 (51.1%)	5789 (32.3%)	4078 (22.8%)	3337 (18.7%)	3128 (17.8%)	6503 (36.3%)
Women	4623 (72.0%)	4458 (69.5%)	3548 (55.2%)	184 (2.8%)	2407 (37.6%)	132 (2.0%)	2818 (43.8%)
*P* value*	**< 0.001**	**< 0.001**	**< 0.001**	**< 0.001**	**< 0.001**	**< 0.001**	**< 0.001**

Data are presented as mean ± SD and number (%)

*Men versus women risk factor distribution difference, statistically significant values are bolded

### Long term outcome

#### Men versus women

**Fig. 2:**
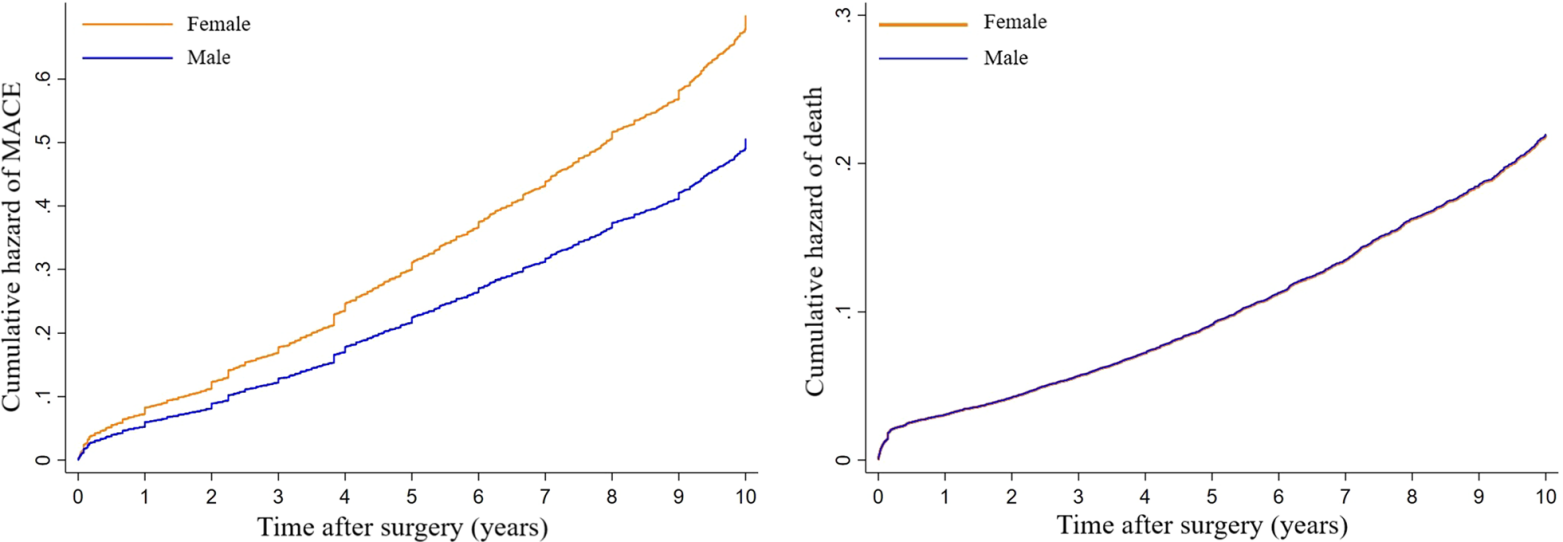
Cumulative hazard of mortality and major adverse cardiovascular events (MACEs) after coronary artery bypass grafting in inverse probability weighting (IPW) model

Men under 50 had the best survival (93.6%) and the least incident of MACE (20.8%). Worst survival was observed in female patients older than 55 (83.4%) who also had the highest incident of MACE (30.6%). In general, patients had 85.9% overall survival and 74.1% of them survived without any MACE (Table 3). Using Cox regression and IPW model (balancing men and women based on their risk factors and comorbidities) we observed that men had lower MACE (HR 0.72; 95% CI 0.57–0.91; *P* value 0.006) but mortality showed no significant difference between sexes (HR 1.00; 95% CI 0.79–1.28; *P* value 0.98) (Table 4, Fig. 2).

**Table 3:** Overall survival and survival without major adverse cardiovascular events after CABG in different age-sex groups

Age-sex category		Overall survival		MACE-free survival	
Men	Men ≤ 50	86.2%	93.6%	75.4%	79.2%
	Men > 50		84.80%		74.70%
Women	Women ≤ 55	84.9%	90.5%	70.3%	73.6%
	Women > 55		83.4%		69.4%

MACE: major adverse cardiovascular event

**Table 4:** Comparison of mortality and major adverse cardiovascular events after CABG between sex groups using Cox regression and inverse probability weighting

Sex	Outcome	Model	HR	CI 95%		*P* value*
				Lower	Higher	
Men versus women	Mortality	Model 1	0.91	0.84	0.98	**0.01**
		Model 2	0.85	0.78	0.93	**< 0.001**
		Model 3	1.00	0.79	1.28	0.98
	MACE	Model 1	0.81	0.77	0.85	**< 0.001**
		Model 2	0.82	0.77	0.87	**< 0.001**
		Model 3	0.72	0.57	0.91	**0.01**

Model 1: Unadjusted Cox regression. Model 2: Multivariate Cox regression for outcome. Model 3: Inverse probability weighting (IPW)

MACE: Major adverse cardiovascular event, HR: hazard ratio, CI: confidence interval

*Statistically significant *P* values are bolded

#### CADRF

As for the association of each risk factor with mortality and MACE, we used adjusted multivariate Cox regression.

In general DM, HTN, and cigarette smoking were associated with significantly higher MACE and DLP and positive family history were associated with lower MACE. The opium addiction effect was only significant in men.

As for mortality, pre-op DM, HTN, and opium addiction led to higher mortality Obesity and opium addiction effect was only significant in the male population (Table 5).

**Table 5:** CADRFs effect on mortality and MACE in all patients and both sexes using multivariate Cox regression

CADRFs	Death				MACE			
	HR	95% CI		*P* value*	HR	95% CI		*P* value*
		Lower	Higher			Lower	Higher	
*All patients*								
Diabetes mellitus	1.49	1.39	1.60	**< 0.001**	1.38	1.31	1.46	**< 0.001**
Hypertension	1.46	1.36	1.58	**< 0.001**	1.31	1.24	1.38	**< 0.001**
Dyslipidemia	0.75	0.70	0.81	**< 0.001**	0.88	0.84	0.93	**< 0.001**
Obesity	0.90	0.82	0.98	**0.011**	1.02	0.96	1.08	0.567
Positive Family History	0.70	0.65	0.76	**< 0.001**	0.87	0.83	0.92	**< 0.001**
Cigarette smoking	1.03	0.93	1.13	0.613	1.08	1.00	1.16	**0.041**
Opium addiction	1.21	1.09	1.34	**< 0.001**	1.13	1.05	1.22	**0.002**
*Women*								
Diabetes mellitus	1.58	1.38	1.82	**< 0.001**	1.38	1.25	1.51	**< 0.001**
Hypertension	1.59	1.35	1.86	**< 0.001**	1.26	1.13	1.40	**< 0.001**
Dyslipidemia	0.77	0.67	0.89	**< 0.001**	0.87	0.78	0.96	**0.006**
Obesity	0.92	0.81	1.06	0.253	1.02	0.93	1.12	0.653
Positive Family History	0.67	0.59	0.77	**< 0.001**	0.86	0.78	0.94	**0.001**
Cigarette smoking	1.04	0.70	1.54	0.853	1.31	1.01	1.70	**0.043**
Opium addiction	1.44	0.97	2.15	0.069	1.18	0.87	1.59	0.29
*Men*								
Diabetes mellitus	1.44	1.32	1.57	**< 0.001**	1.34	1.26	1.43	**< 0.001**
Hypertension	1.42	1.31	1.55	**< 0.001**	1.29	1.22	1.38	**< 0.001**
Dyslipidemia	0.74	0.68	0.80	**< 0.001**	0.88	0.82	0.93	**< 0.001**
Obesity	0.87	0.78	0.97	**0.013**	0.97	0.90	1.05	0.434
Positive Family History	0.71	0.65	0.78	**< 0.001**	0.87	0.82	0.93	**< 0.001**
Cigarette smoking	1.03	0.93	1.15	0.544	1.10	1.02	1.18	**0.017**
Opium addiction	1.21	1.08	1.35	**0.001**	1.17	1.08	1.27	**< 0.001**

*Statistically significant *P* values are bolded

**Fig. 3:**
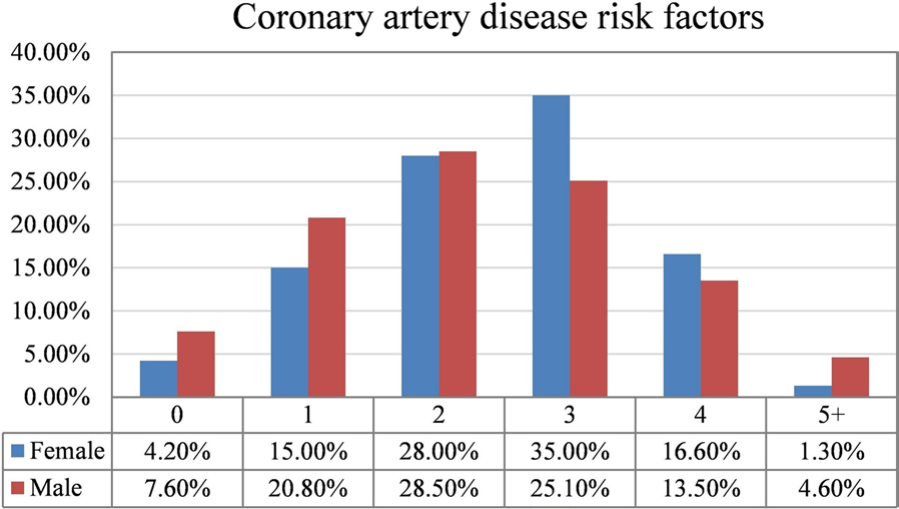
Total risk factor counts between two sexes

Finally, we compared men and women in the number of CADRFs. Near half of the patients had 3 or more risk factors and on average, female patients had slightly more risk factors (Fig. 3).

## Discussion

Based on the results of this registry-based study, patients undergoing CABG are becoming older [12–14]. HTN, DM, obesity, and cigarette smoking increased in nearly all age-sex groups but positive family history and dyslipidemia tend to decrease over the years. LDL level is decreasing which is mainly due to higher statin use in the general population based on previous practice guidelines.

Prevalence of CAD is higher in men before the firth decade of life but during the sixth decade it equalizes and subsequently, it becomes greater in women, Postmenopausal increase in the risk of CAD is related to a higher incidence of hypertension, diabetes, dyslipidemia, and obesity [15]. Menopause affects women in many ways, weight gain with alteration in fat distribution; centripetal obesity and visceral fat deposition are among these changes. Also, with the decrease in estrogen, systolic blood pressure tends to increase and is modulated by the increase in plasma renin activity [16]. All this was in line with our study as we observed that CADRFs except smoking and opium addiction were significantly higher in female patients. The most important clinically relevant finding was higher MACE incidents in women which raise the necessity for better surveillance of CADRFs in female patients.

### Hypertension

In our data registry, hypertensive patients in all 4 subgroups had been decreased until 2012; but started to rise again. An increasing number of hypertensive patients among CABG candidates is in contrast to reports by Esteghamati et al. in 2017 and Azizi et al. in 2019 regarding the prevalence of HTN among the general population of Iran [17, 18]. In our study, HTN was the most frequent risk factor among females older than 55 and is less frequent among males younger than 50 in comparison to other subgroups. Although it is decreasing in the general population, HTN is still a major risk factor for severe CAD and it had a significant effect on post-CABG mortality and MACE. This is an alarming sign for the Iranian population. Preventive and diagnostic measures in the general population as the primary prevention and also precise therapeutic measures for secondary prevention should be taken in hypertensive patients [19].

### Dyslipidemia

DLP which is a well-known CADRFs decreased in our patients who underwent CABG. According to trends of cardiovascular risk factors (based on STEPS reports) in the diabetic and non-diabetic general population in Iran, between 2007–2017, levels of Triglyceride(TG), Low-Density Lipoprotein(LDL) are also decreasing [20]. Increased Statins prescription and general awareness of healthier diets and national primary prevention programs lead to lower lipid profiles in both patients and the general population [21]. Interestingly we found out that DLP before CABG was associated with higher survival and lower MACEs. It was also noted in Yousufuddin et al. study which concluded that in patients with AMI, concomitant HLP was associated with increased survival and a net gain in life years, independent of survival benefit from statin therapy [22].

### Diabetes mellitus

The number of diabetic patients increased from 2007–2017 in all subgroups. It was one of the most prevalent risk factors especially in female patients in both age groups which were present in nearly two-thirds of them and diabetic patients tended to have less fortunate outcomes post-CABG. A similar uptrend is also observed in other studies [23–25]. As for the general Iranian population, DM had an increasing trend with a higher prevalence among women similar to our study [26].

### Family history

As expected, based on the results of our study positive family history was more prevalent among the premature group (Almost half of young men and women had a positive family history of CAD). We also observed that positive family history was higher in women ≤ 55. As an important finding positive family history was associated with lower mortality and MACE post-CABG which was in line with Preisler et al. study [27]. As Ruttmann et al. study suggests this may be because a positive family history as a reason for premature CVD is likely a more benign alternative regarding survival outcomes than some of the other causes that lead to this disease [28].

### Obesity

Obesity (as defined by BMI > 30 kg/m2) was most prevalent in women ≤ 55 and higher in female patients in general. In our study, the trend of obesity was not increased in contrast to other studies [29, 30]. Although obesity is higher among women, an increasing trend is visible among the young male population which is an alarming sign and primary prevention measures should seriously focus on this age group. We also observed the “obesity paradox”, although not associated with MACE, obese patients had better survival. According to Carbone et al., this paradox can be because obesity is defined as an excess fat mass, but individuals with obesity typically also present with an increased amount of lean mass which is associated with improved cardiorespiratory fitness, a major determinant of clinical outcomes [31].

### Cigarette smoking

Cigarette smoking increased in men in both groups. Women had a relatively low prevalence of smoking (under 5%). Our findings are similar to the general Iranian population and probably cultural differences between the two sexes are the main reason for fewer women smokers [32]. The smoking uptrend in the general population and our study may suggest the difficulty of controlling cigarette smoking as one of the major modifiable CADRFs and the necessity of better primary prevention [33].

### Opium addiction

The trend of opium addiction was very similar to cigarette smoking. In contrast to cigarette smoking, there is controversy regarding opium addiction as a CADRF and people usually don’t see it as a serious risk factor for heart problems [34]. We found out that opium addiction is associated with higher mortality ad MACE which is in line with recent studies that show a connection between opium addiction and adverse outcomes in post-CABG patients [35, 36]. In our study, the prevalence of opium addiction is higher in premature CAD patients in both sexes. Hence it is important to increase public knowledge about this modifiable risk factor of CAD in, especially young age group.

Despite the strengths of our study, including large sample size, IPW to reduce the bias of unweighted estimators, and detailed follow-ups years after CABG surgery, we need to mention our limitations. First, due to its observational nature, there are possible inherent biases. Second, this is a single-center study on the Iranian population. Another limitation was that some of the data like cigarette and opium addiction were based on patients’ statements which can lead to reporting bias. Also, we did not separate patients with acute coronary syndrome from a chronic coronary syndrome which might affect our post-CABG survival results.

## Conclusion

Although DLP is still the most frequent CADRF among the CABG population, the level of LDL and TG is decreasing. Diabetes mellitus and hypertension are associated with higher MACE and mortality in both sexes. According to our study, female patients have a higher chance of long-term MACE after CABG surgery. These observations combined with underestimation of CAD in women especially in younger patients can lead to high mortality and morbidity among women with cardiovascular problems. Therefore, health care providers and legislators must pay greater attention to the female population’s CADRFs and ways to prevent them at different levels.

## Data Availability

The data of this study is registered in a database and is available upon request.
